# Clinical practice guidelines for the treatment of extragenital endometriosis in Japan, 2018

**DOI:** 10.1111/jog.14522

**Published:** 2020-10-20

**Authors:** Tetsuya Hirata, Kaori Koga, Kentaro Kai, Hidetaka Katabuchi, Mari Kitade, Jo Kitawaki, Masatoshi Kurihara, Naoko Takazawa, Toshiaki Tanaka, Fuminori Taniguchi, Jun Nakajima, Hisashi Narahara, Tasuku Harada, Shigeo Horie, Ritsuo Honda, Koji Murono, Kotaro Yoshimura, Yutaka Osuga

**Affiliations:** ^1^ Department of Obstetrics and Gynecology Doai Kinen Hospital Tokyo Japan; ^2^ Department of Obstetrics and Gynecology University of Tokyo Tokyo Japan; ^3^ Department of Obstetrics and Gynecology Oita University Oita Japan; ^4^ Department of Obstetrics and Gynecology Kumamoto University Kumamoto Japan; ^5^ Department of Obstetrics and Gynecology Juntendo University Tokyo Japan; ^6^ Department of Obstetrics and Gynecology Kyoto Prefectural University Kyoto Japan; ^7^ Pneumothorax Research Center and Division of Thoracic Surgery Nissan Tamagawa Hospital Tokyo Japan; ^8^ Department of Urology Juntendo University Tokyo Japan; ^9^ Department of Surgical Oncology University of Tokyo Tokyo Japan; ^10^ Department of Surgery International Catholic Hospital Tokyo Japan; ^11^ Department of Obstetrics and Gynecology Tottori University Tottori Japan; ^12^ Department of Thoracic Surgery University of Tokyo Tokyo Japan; ^13^ Department of Plastic Surgery Jichi Medical University Shimotsuke Japan

**Keywords:** bladder/ureteral endometriosis, guidelines, intestinal endometriosis, thoracic endometriosis, umbilical endometriosis

## Abstract

The aim of this publication is to disseminate the clinical practice guidelines for the treatment of intestinal, bladder/ureteral, thoracic and umbilical endometriosis, already published in Japanese, to non‐Japanese speakers. For developing the original Japanese guidelines, the clinical practice guideline committee was formed by the research team for extragenital endometriosis, which is part of the research program of intractable disease of the Japanese Ministry of Health, Labor and Welfare. The clinical practice guideline committee formulated eight clinical questions for the treatment of extragenital endometriosis, which were intestinal, bladder/ureteral, thoracic and umbilical endometriosis. The committee performed a systematic review of the literature to provide responses to clinical questions and developed clinical guidelines for extragenital endometriosis, according to the process proposed by the Medical Information Network Distribution Service. The recommendation level was determined using modified Delphi methods. The clinical practice guidelines were officially approved by the Japan Society of Obstetrics and Gynecology and the Japan Society of Endometriosis. This English version was translated from the Japanese version.

## Introduction

Endometriosis is defined as the presence of endometrium‐like tissue in organs other than the uterus. It has been reported that 5–10% of women of reproductive age experience endometriosis.^1^, ^2^ Endometriosis is an estrogen‐dependent inflammatory disease that causes pelvic pain and infertility. Endometriosis usually occurs in the ovaries, ligaments and peritoneal surface, and less commonly in the intestine, bladder, ureter, abdominal wall, thoracic cavity and other organs.^3^ Extragenital endometriosis is a rare disease for which limited information is available in the literature. As extragenital endometriosis occurs in various organs other than gynecological organs and presents with non‐gynecological symptoms, multidisciplinary collaboration is important for its optimal management and treatment. The clinical practice guidelines was published for the treatment of intestinal, bladder/ureteral, thoracic and umbilical endometriosis in Japan in 2018.^4^ The objective of the guidelines was to enable users to make their practice evidence‐based and to minimize any variations in clinical practice for extragenital endometriosis, thereby facilitating multidisciplinary collaboration. These guidelines were originally written in Japanese, and this English version was translated from the Japanese version.^4^

## Procedure of Developing the Guidelines

The clinical practice guideline (CPG) committee was formed by the research team studying extragenital endometriosis, which is supported by the research program for intractable disease of the Japanese Ministry of Health, Labor and Welfare. The members of CPG committee included experts of gynecology, gastrointestinal surgery, urology, respiratory surgery and plastic surgery. The CPG committee formulated eight clinical questions for the treatment of intestinal, bladder/ureteral, thoracic and umbilical endometriosis, structured by the PICO (P: patients, I: intervention, C: comparisons, O: outcomes) format. For each question, a literature search was performed by the Japan Medical Library Association (http://plaza.umin.ac.jp/~jmla/eng/index_eng.html), a nonprofit organization, using the PubMed, Cochrane and Ichushi databases covering the period from January 2006 to November 2016. The Ichushi database is an Internet‐based retrieval service for Japanese medical article information provided by the NPO Japan Medical Abstract Society. English or Japanese keywords, listed from each PICO formatted clinical questions, were used for the literature search.

The systematic review was performed by an initial and secondary screening of the literature identified by the Japan Medical Library Association. We evaluated and integrated evidence from the systematic review of these articles and wrote the Systematic Review Reports. The strength of the supporting evidence was scored as A (strong), B (moderate), C (weak) or D (very weak), according to the Medical Information Network Distribution Service guideline and the Grading of Recommendation, Assessment, Development, and Evaluation systems.^5^ Furthermore, the recommendations and recommendation levels were established by all members of the CPG committee using modified Delphi methods. The strength of the recommendation was classified as 1 (strong) or 2 (weak). After finalization of the guideline draft, it was submitted to the Japan Society of Obstetrics and Gynecology (JSOG) and the Japan Society of Endometriosis (JSE) for external review and evaluation and for collecting public opinion. The CPG draft was revised accordingly, and the final version was approved by the CPG committee, JSOG and JSE.

## Clinical Questions and Recommendations

The summary of clinical questions and recommendations is shown in Table 1.

**Table 1:** Summary of clinical questions and recommendations

#	Clinical question	Recommendation	Strength of recommendation	Strength of supporting evidence
1	Is medical therapy recommended for intestinal endometriosis?	Medical therapy is recommended for rectosigmoid endometriosis because it is effective in improving symptoms and reducing the size of lesions.	1	C
		The efficacy of medical therapy for intestinal endometriosis other than the rectodigmoid (ileocecal region, appendix, small intestine) is unknown.	2	D
2	Is surgical therapy recommended for intestinal endometriosis?	Surgical treatment is recommended for symptomatic intestinal endometriosis that is difficult to control with medical therapy.	1	C
3	Is medical therapy recommended for bladder endometriosis/ureteral endometriosis?	Medical therapy is effective and recommended for treating bladder endometriosis.	1	C
		Medical therapy may be less effective for ureteral endometriosis with hydroureteronephrosis.	2	C
4	Is surgical therapy recommended for bladder endometriosis/ureteral endometriosis?	Surgical treatment for bladder endometriosis/ureteral endometriosis may be effective depending on the case and operative method.	1	C
5	Is surgical therapy recommended for thoracic endometriosis?	Surgical treatment for catamenial pneumothorax may be effective depending on the symptoms.	1	C
		Catamenial hemoptysis may be ameliorated by conservative treatment without surgery, but surgery is to be considered when symptoms are severe.	2	D
6	Is medical therapy recommended for thoracic endometriosis?	As for thoracic endometriosis, medical therapy, alone or as postoperative adjuvant therapy, may be considered, depending on the case.	2	C
7	Is surgical therapy recommended for umbilical endometriosis?	For umbilical endometriosis, radical surgery with wide local excision is recommended, depending on the case.	1	C
8	Is medical therapy recommended for umbilical endometriosis?	Medical therapy can be an option for umbilical endometriosis.	2	D

Strength of recommendation was classified as 1 (strong) or 2 (weak). Strength of supporting evidence was scored as A (strong), B (moderate), C (weak) or D (very weak).

**CQ 1: Is medical treatment recommended for intestinal endometriosis?**

**Recommendation**:

- Medical therapy is recommended for rectosigmoid endometriosis because it is effective in improving symptoms and reducing the size of lesions. (1C)
- The efficacy of medical therapy for intestinal endometriosis other than the rectosigmoid (ileocecal region, appendix, small intestine) is unknown. (2D)

**Literature search and screening**

For this CQ concerning intestinal endometriosis, 33 English articles and 35 Japanese articles were found by searching the PubMed, Cochrane and Ichushi databases. In the first screening, 17 English articles and 17 Japanese articles were selected. In the second screening, eight English papers and one Japanese paper were used to provide a response to this CQ.

Of the nine studies, one was a systematic review,^6^ seven were prospective cohort studies^7^, ^8^, ^9^, ^10^, ^11^, ^12^, ^13^ and one was a retrospective study.^14^ All articles focused on rectosigmoid endometriosis, and there were no articles concerning endometriosis of the ileocecal region, small intestine or appendix.

**Review and comment**

1. Rectosigmoid endometriosis

With regard to the outcomes, eight studies^6^, ^7^, ^8^, ^9^, ^10^, ^12^, ^13^, ^14^ evaluated symptom and some of these studies^6^, ^10^, ^11^, ^14^ evaluated the lesion size. However, comparisons were not conducted between groups with and without medical therapy, but before and after medical treatment, or between several medicines. In addition, five of the seven prospective cohort studies were from the same institution in Italy,^7^, ^8^, ^9^, ^11^, ^12^ which could represent a bias.

A systematic review of 217 cases from 7 papers reported that 6–12 months of vaginal danazol, GnRH agonist, progestin‐releasing intrauterine system and oral contraceptives (OC) were all effective in relieving pain.^6^ Among seven prospective cohort studies, one evaluated the size of the lesions as an outcome, and reported that 12‐month of norethisterone, GnRH analog plus tibolone add‐back therapy, norethisterone plus letrozole, desogestrel and OC were all effective in reducing the size.^11^ Four studies evaluated the symptoms as an outcome. GnRH analog plus estradiol add‐back therapy for 3 months,^13^ GnRH analog plus tibolone add‐back for 12 months,^9^ norethisterone plus letrozole for 6 months^7^ and norethisterone monotherapy for 12 months^8^ were all effective in improving symptoms, compared to their pre‐treatment conditions. Two studies evaluated both the symptoms and the size of the lesions as outcomes. One study compared a 12‐month estrogen/progestin vaginal ring with orally administered desogestrel, and concluded that both treatments were effective in pain relief and reducing lesion sizes, and that patient satisfaction was significantly higher with oral desogestrel.^12^ The other study reported that continuous administration of OC for 12 months was effective in reducing symptoms and lesions compared to before treatment.^10^

In a retrospective study of 82 cases of intestinal endometriosis, 55 patients (67.1%) underwent hormonal therapy (OC, dienogest, GnRH agonist or danazol), and 43 cases (78.2%) showed improvement in symptoms.^14^ In particular, dienogest was highly effective in reducing lesion sizes and improving symptoms.

Although no detailed studies have been conducted examining adverse effects, none of the articles reported any serious adverse effects or complications. Note that almost all these reports examined the effect of medical therapy within 1 year, and no report to date has examined the long‐term prognosis such as the recurrence rate after discontinuation of the therapy.

2. Intestinal endometriosis of the ileocecal region, small intestine, and appendix

A review of the literature revealed that a few case reports and case series have been published and there are currently no reports assessed on the efficacy of medical treatment for these rare types of intestinal endometriosis.

**Summary**

For rectosigmoid endometriosis, medical therapy may be useful for improving symptoms and reducing lesion sizes. Given the high complication rate of surgery, medical treatment may be the first option, although there is no evidence of superiority to surgery. For intestinal endometriosis other than for rectosigmoid (e.g., ileocecal, appendix, small intestine), there is no available evidence of the efficacy of medical treatment, or its superiority to study. Medical therapy can be an option for this rare type of intestinal endometriosis; however, when the symptoms are not controlled by medical treatment, surgical therapy should be considered.

The adverse effects and complications of medical therapy for intestinal endometriosis may be comparable to those used for pelvic endometriosis, such as ovarian endometrioma, although unique complications of medical therapy when used for intestinal endometriosis are unknown. The recurrence rate after discontinuation of medication is also unknown, but long‐term management may be required, as in the case of endometriosis at other sites.

**CQ 2: Is surgical therapy recommended for intestinal endometriosis?**

**Recommendation:**

- Surgical treatment is recommended for symptomatic intestinal endometriosis that is difficult to control with medical therapy. (1C)

**Literature search and screening**

For surgical treatment of intestinal endometriosis, 246 English and 251 Japanese articles were identified by searching the PubMed, Cochrane and Ichushi databases. At the first screening of these publications, 123 English papers and 5 Japanese papers were selected, and in the second screening, 19 English papers were selected to answer this CQ.

Of these 19 studies, 13 were case series,^15^, ^16^, ^17^, ^18^, ^19^, ^20^, ^21^, ^22^, ^23^, ^24^, ^25^, ^26^, ^27^ 4 were cohort studies^28^, ^29^, ^30^, ^31^ and 2 were randomized controlled trials (RCT).^32^, ^33^

**Review and comment**

The four cohort studies^28^, ^29^, ^30^, ^31^ and the two RCT^32^, ^33^ compared different surgical methods, and did not compare a surgically treated group with a non‐treated group. Five case series,^15^, ^16^, ^17^, ^18^, ^19^ 1 cohort study (ablative surgery with [study group] or without bowel resection [control group]),^28^ and one RCT (laparotomy vs laparoscopic surgery) examined postoperative improvement of symptoms.^32^ In all studies, surgical treatment showed improvement in symptoms.

Regarding surgical complications, three cohort studies^28^, ^30^, ^31^ and two RCT^32^, ^33^ did not report the details of the complications; thus, the frequency of serious complications such as anastomotic insufficiency and rectovaginal fistula are unknown. Eleven case series^15^, ^16^, ^17^, ^18^, ^20^, ^21^, ^22^, ^23^, ^24^, ^25^, ^26^ and one cohort study (nerve‐sparing surgery vs conventional surgery)^29^ described postoperative complications in detail.

In four case series reports,^15^, ^16^, ^17^, ^18^ the authors examined postoperative symptom improvement. In one case series,^19^ one cohort study^28^ and one RCT^32^ symptom improvement was evaluated by scoring. All studies suggested that surgical treatment improved symptoms. Therefore, although the level of evidence is low, surgical treatment may improve symptoms.

With regard to complications, the frequency of serious complications such as anastomotic insufficiency, rectovaginal fistula and dysuria was investigated in 11 case series,^15^, ^16^, ^17^, ^18^, ^20^, ^21^, ^22^, ^23^, ^24^, ^25^, ^26^ and in 1 cohort study.^29^ Anastomotic insufficiency, rectovaginal fistula and dysuria were reported in 0–2%, 1.8–4% and 0.8–29% of cases, respectively. Since serious complications have been reported, the indications for surgery should be defined considering the risk of complications.

**Summary**

Among the 19 papers reviewed, there were no reports comparing surgically treated cases with non‐surgically‐treated cases, and there is no literature available that directly answered this CQ. However, according to the literature, surgical treatment can improve or relieve symptoms. Therefore, surgical therapy is an option for symptomatic intestinal endometriosis that is difficult to control with other therapies.

**CQ 3: Is medical therapy recommended for bladder endometriosis/ureteral endometriosis?**

**Recommendation:**

- Medical therapy is effective and recommended for treating bladder endometriosis. (1C)
- Medical therapy may be less effective for ureteral endometriosis with hydroureteronephrosis. (2C)

**Literature search and screening**

We identified 19 English articles and 22 Japanese articles investigating medical therapy for bladder endometriosis and ureteral endometriosis by searching the PubMed, Cochrane and Ichushi databases. Eleven English articles and eight Japanese articles (all English articles were case reports and case series) were selected for the primary screening. In the secondary screening, four English articles (one systematic review, two reviews and one case series) were selected as the final references. One systematic review included five English papers. Of the four English papers, two were about bladder endometriosis^34^, ^35^ and one was a review of ureteral endometriosis.^36^ These reviews included case reports and case series excluded by screening.

There were no RCT, meta‐analyses or cohort studies. There was one report of ureteral endometriosis treated with aromatase inhibitors,^37^ but it was not included in the above three articles and was added to the final article list.

**Review and comment**

1. Bladder endometriosis

In a systematic review of the literature on bladder endometriosis, 36 patients were included in 9 case reports describing medical therapy.^34^ The authors suggested that OC and dienogest were the first choice treatments, and GnRH agonists were the second choice, both of which require long‐term treatment. Administration of dienogest for 6 months or more had the effect of reducing lesions and improving symptoms. It has also been pointed out that GnRH agonists have a stronger effect on lesion reduction than OC. Considering that aromatase inhibitors are often ineffective, have side effects such as muscle pain and joint pain, and are still a focus of clinical research, they should be considered as an option when the first and second choice treatments are ineffective. It has been pointed out that all medical therapies have a temporary effect during treatment.

Another article reviewed 23 studies on medical therapy for bladder endometriosis reported between 1996 and 2011.^35^ In addition to the GnRH agonists, dienogest and OC, this review suggested the efficacy of medal progesterone acetate depot and danazol treatment.

Medical therapy was most suitable for patients with small endometriotic lesions of ≤5 mm in size, but long‐term administration is required because the recurrence rate after discontinuation of medication is high at 35%.

Ureteral endometriosis

One article reviewed two reports on medical therapy for ureteral endometriosis reported from 1996 to 2010.^36^ The review showed that the course of hormonal therapy may be similar to that of bladder endometriosis, that medical treatment is effective, and that ultrasound examination every 6 months could assess the development of ureteral obstruction during treatment. Hormone therapy appears to be effective in suppressing the growth of ureteral endometriotic tissue, but has modest effects on fibrotic lesions and scars. Furthermore, it has been pointed out that hormonal treatment alone has a poor effect on lesions with ureteral obstruction due to adhesion with surrounding tissues. In addition, there was a case report describing a patient with bilateral ureteral endometriosis receiving aromatase inhibitor.^37^ Another report describing a patient who received a GnRH agonist for 6 months, and subsequently an aromatase inhibitor for 15 months, reported that fibrotic lesions were not reduced, and the patient eventually required surgical treatment.

**Summary**

For patients with bladder endometriosis, medical therapy can be the first choice because it can be expected to relieve symptoms. For ureteral endometriosis, the same medical therapy for bladder endometriosis may be given. However, when ureteral stenosis is caused by a fibrotic lesion, the therapeutic effect is poor. Therefore, it is necessary to carefully select suitable patients for treatment. In any urinary tract endometriosis, medical treatment can be expected to relieve symptoms and reduce lesions, but long‐term therapy is required.

**CQ 4: Is surgical therapy recommended for bladder endometriosis/ureteral endometriosis?**

**Recommendation:**

- Surgical treatment for bladder endometriosis/ureteral endometriosis may be effective depending on the case and operative method. (1C)

**Literature search and screening**

First, for this CQ concerning bladder/ureteral endometriosis, 228 English documents and 122 Japanese documents were searched using the PubMed, Cochrane and Ichushi databases. A total of 102 English papers were selected for the primary screening, and 20 English papers were selected for this CQ in the secondary screening.

No RCT has examined the efficacy of surgical therapy. Of the 20 articles identified, 4 were prospective case series^38^, ^39^, ^40^, ^41^ and only 1 was a prospective study with comparison of surgical treatment.^38^ Sixteen studies retrospectively summarized surgical cases for bladder and ureteral endometriosis,^42^, ^43^, ^44^, ^45^, ^46^, ^47^, ^48^, ^49^, ^50^, ^51^, ^52^, ^53^, ^54^, ^55^, ^56^, ^57^ most of which involved laparoscopic surgery and one involved robot‐assisted surgery.^45^

**Review and comment**

To answer this CQ, a comparative study of surgical treatment and non‐surgical treatment is necessary. In addition, prospective RCT are essential because of the potential impact of various factors such as the severity of endometriosis, patient age, symptoms, and pretreatment administered. The answer depends greatly on the criteria defined as ‘effective’. For example, postoperative recurrence rate, reoperation rate, complications, alleviation of symptoms, improvement of reproductive function, improvement of urinary function, improvement of hydronephrosis, and so on are assumed to be the criteria of ‘effectiveness of surgical treatment’. However, no valid results have been reported for these studies.

Generally, surgical treatment for bladder endometriosis/ureteral endometriosis includes ureterolysis, ureteroureterostomy, ureterocystoneostomy, transurethral resection of endometriosis,^54^ partial cystectomy and nephrectomy, each of which may be performed by open surgery, endoscopic surgery, or robotic‐assisted surgery. In addition, ureteroscopic ablation with a holmium YAG laser has also been reported.^46^

In a prospective case series of 56 cases of ureteral endometriosis, Mereu *et al*. performed laparoscopic ureterolysis (35 cases), laparoscopic ureteroureterostomy (17 cases), laparoscopic ureterocystoneostomy (2 cases) and laparoscopic nephrectomy (2 cases).^38^

Comparing the 35 cases of laparoscopic ureterolysis and 17 cases of laparoscopic ureteroureterostomy, ureterolysis cases presented significantly more complications, during a median follow‐up period as short as 21 months. Soriano *et al*. reported that among 41 of 45 patients (91.1%) that underwent laparoscopic ureterolysis, only 2 (4.4%) needed reoperation.^39^ In addition, Seracchiolo *et al*. reported that 22 of 30 patients (73.3%) underwent ureterolysis and 8 patients (26.7%) had recurrence of endometriosis during the average follow‐up period of 55 months.^41^ As described above, the evaluation of efficacy may vary greatly depending on the type of surgical therapy. If limited to ureteral endometriosis, ureteral stent placement is a surgical treatment option and needs to be evaluated.

Accordingly, there are variations in the requirements for the selection of surgical procedures for bladder endometriosis and ureteral endometriosis, and thus the results may be biased. The choice of surgical therapy and the actual surgical procedure are strongly affected by the patient's age, clinical condition (particularly reproductive and renal function), or the stricture site and extent of urinary tract involvement.^39^ There is not enough evidence to respond to this CQ, as there are no studies comparing surgery to other therapies and only results from surgery alone have been reported. However, surgery may be effective because there are consistent reports of symptom improvement and low postoperative recurrence rate in case series. Further studies are also needed on the effectiveness of medical treatment as a prior treatment/combination therapy. In the future, it would be desirable to establish an algorithm for selecting treatment methods, including surgical therapy.

**Summary**

For this CQ, there is no clear evidence from case series reports. However, considering reports of each case series, surgical treatment for bladder endometriosis/ureteral endometriosis may be effective, and further study is required to provide an answer to this CQ.

**CQ 5: Is surgical therapy recommended for thoracic endometriosis?**

**Recommendation:**

- Surgical treatment for catamenial pneumothorax may be effective depending on the symptoms. (1C)
- Catamenial hemoptysis may be ameliorated by conservative treatment without surgery, but surgery is to be considered when symptoms are severe. (2D)

**Literature search and screening**

To determine the efficacy of surgical treatment of thoracic endometriosis, 75 articles in English were identified by searching the PubMed, Cochrane and Ichushi databases. Twenty‐four English articles were selected during the first screening, and 13 articles in English were selected as references for this CQ in the second screening. Among these 13 articles, 11 were on catamenial pneumothorax,^58^, ^59^, ^60^, ^61^, ^62^, ^63^, ^64^, ^65^, ^66^, ^67^, ^68^ 1 was on catamenial hemoptysis^69^ and another described both conditions.^70^ One was a multicenter study investigating catamenial hemoptysis,^70^ 10 studies were reports of single‐center case studies of thoracic endometriosis,^58^, ^59^, ^60^, ^61^, ^62^, ^63^, ^64^, ^65^, ^66^, ^70^ and 2 were reviews of previous articles on catamenial pneumothorax.^67^, ^68^ Nine articles on catamenial pneumothorax were retrospective observational studies including 4–150 cases,^58^, ^59^, ^60^, ^61^, ^62^, ^63^, ^64^, ^65^, ^66^ 4 of which revealed the frequency of catamenial pneumothorax among spontaneous pneumothorax in women.^58^, ^61^, ^63^, ^64^ On the other hand, catamenial hemoptysis is a rare disease, and approximately 40 cases have been reported in English. The 19 cases reported in a single multicenter study described the clinical features, imaging findings, treatment and surgical outcomes of catamenial hemoptysis, but there was no comparison with other treatments.^69^ The literature discussing surgical treatment of catamenial pneumothorax described its clinical features, operative procedure and hormonal treatment, and evaluated the pneumothorax recurrence rate as the outcome. Two reviews were on catamenial pneumothorax,^67^, ^68^ and one article discussed the significance of serum CA 125 levels as a diagnostic approach.^66^

**Review and comment**

As a surgical method for spontaneous pneumothorax, it is common to thoracoscopically suture or resect the lesions of the visceral pleura that causes air leakage. Even in the case of catamenial pneumothorax, in the acute phase, the endometriotic lesion that causes air leak should be resected to stop the air leak. In addition, if there are defects in the diaphragm, which are suspected to be the cause of the inflow of the endometrium into the thoracic cavity, it should be closed. Although pleurodesis may be performed to prevent recurrence of pneumothorax due to the onset of pleural lesions, it is not clear whether pleurodesis should be performed. The recurrence rate was defined as the outcome of the case series described in 10 reports of catamenial pneumothorax.^58^, ^59^, ^60^, ^61^, ^62^, ^63^, ^64^, ^65^, ^66^, ^68^ The details of the surgical procedure were described in seven reports,^58^, ^60^, ^62^, ^64^, ^65^, ^66^, ^70^ and postoperative hormonal therapy was described in five reports.^58^, ^61^, ^62^, ^65^, ^70^ None of the articles compared surgical therapy with other treatments and did not provide a clear answer to this CQ.

**Summary**

Of the 13 papers reviewed for thoracic endometriosis, there was no evidence available to answer to this CQ. The clinical features, histopathological findings and postoperative recurrence rate of catamenial pneumothorax and hemoptysis were analyzed in 12^58^, ^59^, ^60^, ^61^, ^62^, ^63^, ^64^, ^65^, ^66^, ^67^, ^68^, ^70^ and 2 case series reports,^69^, ^70^ respectively. If catamenial pneumothorax is very severe, it may cause respiratory failure and may be life‐threatening. Therefore, it is recommended to determine the surgical indication based on spontaneous pneumothorax. For catamenial hemoptysis, surgical therapy may be considered if the condition is severe to cause airway obstruction due to a clot.

**CQ 6: Is medical therapy recommended for thoracic endometriosis?**

**Recommendation:**

- As for thoracic endometriosis, medical therapy, alone or as postoperative adjuvant therapy, may be considered, depending on the case. (2C)

**Literature search and screening**

For this CQ, 111 English and 15 Japanese studies were identified from the PubMed, Cochrane, and Ichushi databases. Eighteen English articles and eight Japanese articles were selected for the primary screening, and eight English articles and three Japanese articles were selected as references for this CQ in the second screening. Of these 11 articles, 10 were case series^65^, ^70^, ^71^, ^72^, ^73^, ^74^, ^75^, ^76^, ^77^, ^78^ and 1 was a retrospective cohort study.^62^ Seven reports were on catamenial pneumothorax,^65^, ^72^, ^73^, ^74^, ^76^, ^77^, ^78^ three were on treatment experiences for thoracic endometriosis^62^, ^70^, ^71^ and one was related to adverse effects of medical therapy.^75^ Of the case series associated with medical therapy, surgical intervention was performed in all cases series except for one report, and even in this remaining one,^70^ there was no comparison between outcomes of medical therapy alone group and the control group. One cohort study investigated on pleurodesis and on the recurrence of catamenial pneumothorax after the treatment with or without postoperative adjuvant hormonal therapy.^62^

**Review and comment**

Ten reports^65^, ^70^, ^71^, ^72^, ^73^, ^74^, ^75^, ^76^, ^77^, ^78^ were reviews of a large number of cases treated for thoracic endometriosis and did not compare the medical treatment group including pleurodesis with the control group. Regarding the recurrence of clinical symptoms after treatment, one paper reported cases treated pharmacologically (GnRH agonist alone or GnRH agonist + dienogest) or surgically,^70^ and eight papers reported cases treated with or without postoperative hormonal therapy,^65^, ^71^, ^72^, ^73^, ^74^, ^76^, ^77^, ^78^ two of which included cases of recurrence after surgery with postoperative dienogest therapy.^76^, ^77^ One was a case series reporting the adverse effects of medical therapy.^75^ No study investigated the effects of medical treatment, including pleurodesis, on the recurrence of catamenial pneumothorax. Therefore, the available evidence is not sufficient to answer this question. One retrospective cohort study reported recurrence of symptoms with and without pleurodesis and postoperative adjuvant drug therapy (GnRH agonist alone), but no control group was used.^62^ None of the reports described the association between lesion size and the effects of medical therapy.

**Summary**

Of the 11 articles reviewed, there was no available literature that provided evidence to directly answer this CQ. However, GnRH agonist alone or GnRH agonist–dienogest sequential administration may be considered as effective medical therapy.

**CQ 7: Is surgical therapy recommended for umbilical endometriosis?**

**Recommendation:**

- For umbilical endometriosis, radical surgery with wide local excision is recommended, depending on the case. (1C)

**Literature search and screening**

A literature search of the PubMed, Cochrane and Ichushi databases retrieved 107 English papers and 32 Japanese papers. In the first screening of these documents, 47 English and 19 Japanese studies were selected, and in the second screening, 13 Japanese^79^, ^80^, ^81^, ^82^, ^83^, ^84^, ^85^, ^86^, ^87^, ^88^, ^89^, ^90^, ^91^ and 33 English^92^, ^93^, ^94^, ^95^, ^96^, ^97^, ^98^, ^99^, ^100^, ^101^, ^102^, ^103^, ^104^, ^105^, ^106^, ^107^, ^108^, ^109^, ^110^, ^111^, ^112^, ^113^, ^114^, ^115^, ^116^, ^117^, ^118^, ^119^, ^120^, ^121^, ^122^, ^123^, ^124^ were selected as relevant articles for this CQ.

Since there has been no prospective comparative study on the efficacy of treatment for umbilical endometriosis, we examined case reports and case series.

**Review and comment**

Of 89 cases of umbilical endometriosis, 82 were treated with surgical therapy and 7 were conservatively treated with medical therapy alone. Considering that the symptoms were improved by surgery, and no specific complications were reported, surgical treatment may be effective in improving symptoms. In most cases, the postoperative follow‐up period was less than 6 months or was not specified. Recurrent patients had relapsed more than 2 years after surgery. Therefore, surgical treatment may be effective in the short term, but the long‐term recurrence rate and complications remain unknown.

In order to avoid recurrence after surgical therapy, it is preferable to excise the lesion to include a large portion of marginal tissue (wide local resection). Umbilical deformities associated with wide local resection may occur and sometimes require umbilical reconstruction by plastic surgeons. Accordingly, surgical therapy may be effective in improving or relieving symptoms, at least in the short term.

Saito *et al*. reported seven cases of umbilical endometriosis, in which three patients were followed up without treatment, three were treated with OC and one was treated with surgery.^113^ Their results suggested that OC treatment is effective in alleviating symptoms. It can be recommended for patients with mild symptoms who do not wish to get pregnant. Conversely, discontinuation of medication often causes relapse of symptoms, and surgical treatment is still necessary as radical treatment.

The etiology of umbilical endometriosis is still unclear, but two types have been described. The first is a secondary umbilical endometriosis that arises on scar tissue following abdominal surgery such as laparoscopic surgery, and the other is primary umbilical endometriosis that occurs in the absence of surgical history. Of the 89 cases of umbilical endometriosis described, 16 cases were secondary and 73 cases were primary. Although there is no literature comparing the therapeutic effects of surgical treatment for primary and secondary umbilical endometriosis, it may possibly affect the therapeutic outcome.

**Summary**

A review of the literature suggests that surgical treatment may have short‐term efficacy for umbilical cord endometriosis, but long‐term efficacy and complications remain unknown. Although no study has directly compared medical therapy and surgical therapy, surgical therapy should be the first choice since the umbilicus is easy to approach and the invasiveness of surgery is relatively minimal.

**CQ 8: Is medical therapy recommended for umbilical endometriosis?**

**Recommendation:**

- Medical therapy can be an option for umbilical endometriosis. (2D)

**Literature search and screening**

Seven English and 10 Japanese articles were identified for the CQ of umbilical endometriosis. Seven English and eight Japanese studies were selected in the first screening, and seven English papers were selected as the final references in the second screening. All seven papers were case reports, two of which included case reports and a review of previously reported cases.^124^, ^125^

**Review and comment**

All reports except one were case reports describing surgical treatment, and the description of the effect of medical therapy was limited. Since surgical treatment is the major treatment for umbilical endometriosis, there has been no literature evaluating the efficacy of medical therapy alone.

In one case series, medical therapy was reported to be effective in improving symptoms in three cases, but the case series was not a study comparing with/without medical therapy or different agents.^113^ Therefore, it is difficult to answer not only the efficacy of medical therapy alone, but also comparison with surgical therapy, comparison between different medicines and complications.

In a review of 231 reported cases, only 3 received medical therapy alone and 228 underwent surgical treatment.^124^ The authors noted that although medical therapy is useful for relieving symptoms, the administration of medical therapy is likely to be prolonged and the disease may recur after discontinuation of medication. Before choosing medical therapy, it is necessary to exclude malignant diseases. In a case series of seven patients, an OC was administered to three patients, and their symptoms improved.^113^ Although symptoms recurred when hormone therapy was discontinued, hormone therapy has been reported to improve symptoms and can be an adjunctive postoperative treatment to prevent recurrence, if the endometriotic lesions are positive for estrogen and progesterone receptor expression.^118^

According to another study, hormone therapy is less effective for endometriosis that develops in the abdominal wall or that arises from surgical scar tissues, and symptoms tend to relapse when the medication is discontinued, but medical therapy is an option if concurrent severe pelvic endometriosis is present.^102^ In addition, preoperative administration of GnRH agonists is not recommended because of the increased risk of incomplete resection due to reduced tumor size and difficulty in its localization. In another study, approximately 70% of umbilical endometriosis was treated surgically, and hormonal therapy may have been effective in reducing tumor size and improving symptoms, although the effect was not certain.^101^ Furthermore, preoperative treatment with dienogest and OC may be effective, but it is difficult to cure the endometriosis with hormone treatment alone, while surgical treatment is preferable to conservative treatment because of complete surgical excision and histological confirmation.^96^

None of the available literature has reported any serious adverse effects or complications of medical therapy.

**Summary**

The reported number of cases of umbilical endometriosis was small, and there were no high‐level evidence articles. Based on the literature review, surgical treatment is the main treatment for umbilical endometriosis, and few cases have received medical therapy. Therefore, it was difficult to answer this CQ. Furthermore, there have been no studies comparing medical and surgical therapy, comparing different agents, reporting the frequency of adverse effects/complications, or the recurrence rate after treatment discontinuation. Further studies are required to answer this CQ.

## Disclosure

None declared.
